# Determinants of physical activity behavior among older adults with subjective cognitive decline based on the capability, opportunity, motivation, and behavior model: mediating and moderating effects

**DOI:** 10.3389/fpubh.2023.1338665

**Published:** 2024-01-08

**Authors:** Yiping Chen, Wei Li, Hui Yang

**Affiliations:** ^1^School of Nursing, Shanxi Medical University, Taiyuan, Shanxi, China; ^2^International Medical Department, Peking Union Medical College Hospital (CAMS), Beijing, China

**Keywords:** physical activity, older adults, subjective cognitive decline, factors, cross-sectional study

## Abstract

**Background:**

PA is vital for secondary prevention in older adults with subjective cognitive decline (SCD), but their physical activity (PA) levels are low, and the underlying interaction pathways among associated factors are poorly understood. This study aims to identify mediating and moderating effects of determinants on PA behavior in older adults with SCD using the capability, opportunity, motivation, and behavior model.

**Methods:**

Following the STROBE checklist, we conducted a cross-sectional survey among 289 older adults with SCD. Path regression, mediation effects, and moderation effects were used to explore the associated factors of PA behavior and the associations among these variables.

**Results:**

The prevalence of physical inactivity among older adults with SCD was high (40.3%). The path model fit indices were χ^2^/df = 1.145, GFI = 0.968, CFI = 0.988, and RMSEA = 0.022. Path regression revealed that frailty, physical and social support, PA motivation, and fall history had significant direct association with PA behavior. PA motivation not only partially mediates between frailty and PA behavior but also partially mediates between physical and social support and PA behavior. Additionally, fall history moderated the relationship between frailty and PA behavior.

**Conclusion:**

PA behavior in older adults with SCD requires improvement. Healthcare professionals should devise more effective interventions to boost PA behavior by enhancing motivation. Screening for frailty and addressing fall history, while providing sufficient physical and social support, is crucial.

## Introduction

Alzheimer's disease (AD) dementia is a major neurodegenerative condition impacting older adults, with increasing prevalence due to global aging trends (1). Despite its significance, effective methods for prevention and treatment remain elusive, emphasizing the importance of early intervention during pre-dementia stages such as subjective cognitive decline (SCD) (2). SCD, characterized by self-reported cognitive impairment without meeting mild cognitive impairment criteria, offers an optimal window for intervention, with a significant prevalence in older populations. Early diagnosis and intervention in the SCD stage are crucial for potentially reversing or delaying progression to AD dementia, thereby reducing the overall impact on individuals, families, and healthcare systems (3, 4).

In the absence of effective pharmacological options to delay SCD progression to AD dementia, non-pharmacological approaches, particularly physical activity (PA), have gained prominence (5). PA has been identified as the most significant modifiable risk factor for dementia, with extensive research indicating its potential in preventing cognitive decline and reducing dementia risk in both primary and secondary prevention contexts (6). As such, PA is recognized as a critical method for reducing the risk of AD dementia in older adults with SCD.

However, older adults with SCD often exhibit lower levels of PA, and research specifically exploring their PA behavior is limited. Theoretical frameworks like the capability, opportunity, motivation, and behavior (COM-B) model (7) offer valuable insights into understanding and promoting PA behavior in this group. This study aims to use the COM-B model to investigate the determinants of PA behavior in older adults with SCD, focusing on key variables such as frailty, physical and social support, PA motivation, and history of falls. By exploring the pathways and interactions of these variables, the study seeks to enhance understanding and inform effective interventions for promoting PA in this population.

## Conceptual framework

A recent umbrella review (8) organized the influencing factors of PA behavior in older dementia/MCI patients based on the theoretical domain framework (TDF), which is considered a useful tool for identifying behavioral determinants and barriers to behavior change. TDF elaborates on the COM-B model, representing “capability,” “opportunity,” “motivation,” and “behavior”. In this review, environmental factors were identified as the highest-rated evidence factors influencing PA behavior in older dementia/MCI patients, followed by social influences, which, based on the COM-B model, can be merged into opportunity factors. The impact of opportunity factors on PA behavior has been validated in multiple previous studies, with opportunity factors typically representing physical and social support for PA behavior. There is evidence that physical support, such as specific elements of the built environment, contributes to promoting PA among older adults (9). Another review study suggests that specific social support for PA is positively correlated with PA levels among the older adult population. Therefore, this study assumes a significant association between physical and social support and PA behavior in older adults with SCD.

In terms of capability, it mainly involves participants' gait characteristics and disease features. Furthermore, previous research has found a strong association between frailty and patients' gait characteristics and PA (10), so in this study, we will use frailty as a variable to measure the capability of PA in older adults with SCD. We assume that frailty is associated with the physical activity behavior of the older adults with SCD. Also, it is worth noting that multiple studies (11, 12) indicate that patients with a history of falls are less likely to engage in PA, which also undermines their confidence in their PA capability. This suggests that whether a fall has occurred may be a moderating factor in the relationship between frailty and PA behavior in older adults with SCD.

Motivation is an important factor that promotes PA behavior. Unfortunately, previous research on motivation for PA behavior in older adults has been largely qualitative (13), lacking quantitative measures. Motivation is an internal drive originating from within the individual, which can initiate, sustain, and regulate behavior. Motivation for PA behavior refers to the internal needs that drive individuals to initiate, maintain, and regulate PA behavior. Therefore, PA motivation is closely related to the PA behavior of older adults. In this study, we hypothesize that it serves as a shared mediating factor between frailty and PA behavior, as well as between physical and social support and PA behavior.

**Figure 1:**
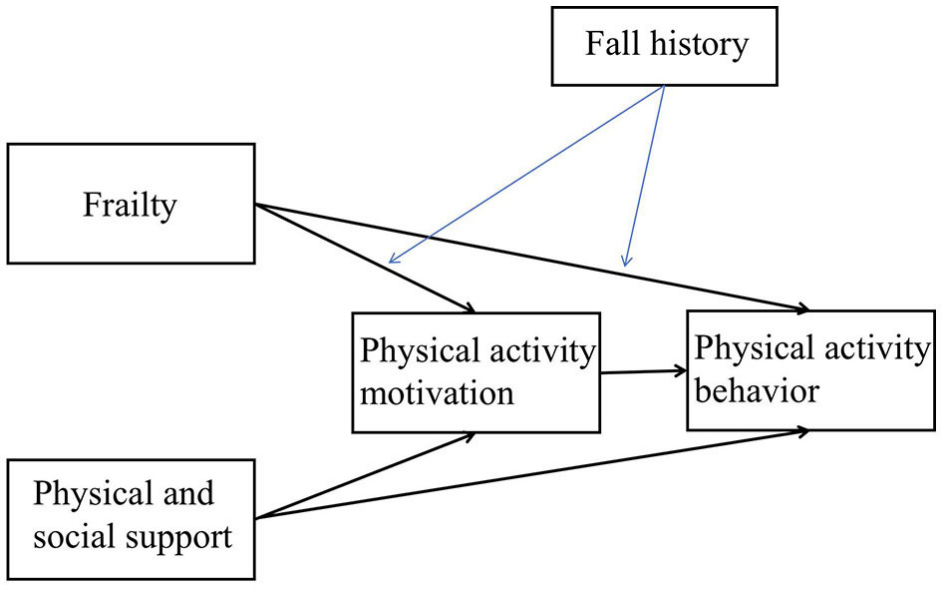
Constructed model for hypothesis testing.

Using theory to identify determinants of behavior can enhance the effectiveness of interventions (7, 14). Based on the COM-B model, this study aimed to describe the related factors of PA behavior of the older individuals with SCD and establish a structural model of capability, opportunity, motivation and PA behavior. This study aimed to elucidate whether frailty have both direct and indirect associations (through PA motivation) with PA behavior; physical and social support have both direct and indirect associations (through PA motivation) with PA behavior; and whether fall history moderates the direct and indirect association between frailty and PA behavior. Figure 1 shows our constructed model for the hypothesis testing.

## Methods

### Study design

This is a cross-sectional survey. The Strengthening the Reporting of Observational Studies in Epidemiology (STROBE) statement was used to report this observational study (see Supplementary material 1).

### Participants and sample size

The survey study was conducted in a county town in Southeastern Fujian Province, which is the hometown of the first author. As the survey was conducted within a single county town, where cultural customs are similar, there is not much heterogeneity in the data. This county town is characterized by the migration of its young and middle-aged labor force and an aging population trend. In the latest census, the population aged 60 and above was 82,859, accounting for 19.61%. Our research team successively contacted the directors of the residents' committees of 15 communities within the town. We requested their assistance in distributing notices about participating in the questionnaire survey, especially inviting older adults to the special senior activity rooms set up in their respective community committees to facilitate the completion of the questionnaires. In the end, the directors of the residents' committees of 12 communities agreed to cooperate with our research. As a token of appreciation for the participants, each older person who completed the SCD screening received a free blood pressure measurement service and a small gift—a pack of tissues. Additionally, those older individuals who met our study inclusion criteria and completed the questionnaire were further compensated with a red envelope worth 50 RMB. These incentive measures were designed to encourage more older people to actively participate in our research and also to show our respect for their valuable time and contributions.

This study employed a combination of convenience sampling and snowball sampling methods for participant recruitment. Researchers conducted subjective cognitive decline screening and MMSE scale assessments on these older adult participants. Inclusion criteria for participants were as follows: (1) age 60 years or older; (2) meeting the criteria for subjective cognitive decline (i.e., The Subjective Cognitive Decline 9-item (SCD-9) questionnaire score > 5 (15) and The Mini-Mental State Examination scale score ≥ 27); and (3) the ability to communicate in Mandarin or a local dialect. Exclusion criteria were as follows: (1) participation in psychosocial interventions during the study period; (2) severe disability; and (3) severe liver or kidney disease or any known malignancy.

The sample size for this study was determined based on established guidelines for structural equation models. It is widely recognized that a minimum of 10–20 participants per observed variable is necessary for structural equation analysis (16). In this study, we designated 15 participants for each observed variable. There are 12 observed variables in this study, including age, gender, marital status, place of residence, educational attainment, current smoking status, current alcohol consumption, and personal monthly income. Additionally, a 10% rate of invalid questionnaires was taken into consideration, leading to a final minimum sample size of 198 participants. Throughout the survey process, the study initially screened 360 older adult individuals who self-reported memory decline using SCD-9 questionnaire. Among them, 305 older adult individuals met the criteria for SCD. Ultimately, 289 older adult individuals with SCD completed the questionnaire survey, with no invalid questionnaires identified (Response rate: 94.8%). Participants took ~20–30 min to complete the questionnaire. Completed questionnaires were collected on-site.

## Measures

### Socio-demographic characteristics

The self-reported personal information form aims to collect socio-demographic data, including age, gender, marital status (married/living with partner or unmarried/widowed), place of dwelling (rural/urban), educational attainment (elementary school or below, middle school, high school or above), current smoking status (yes/no), current alcohol consumption (yes/no), and personal monthly income.

### Physical activity behavior

A modified version of the International Physical Activity Questionnaire (IPAQ) was used to assess participants' physical activity levels in our study, which was widely used in the China Health and Retirement Longitudinal Study (CHARLS) questionnaire (17), specifically on pages 70–71. Participants were asked to report their regular physical activity on a weekly basis using the IPAQ. Physical activity was categorized into three groups: vigorous physical activity (VPA), moderate physical activity (MPA), and light physical activity (LPA). Various aspects of physical activity, such as intensity, duration, frequency, and volume, were measured. According to the World Health Organization (2020) guidelines (18), 1 min of vigorous activity is considered equivalent to 2 min of moderate-intensity activity. To meet the recommended physical activity standards, individuals needed to engage in at least 150 min of moderate-intensity activity per week or a total of at least 75 min of vigorous activity per week. In this study, we categorized the physical activity behavior of older adults with SCD as a binary variable. Older adults with SCD who met the WHO physical activity recommendations were defined as physically active, while those who did not meet the standards were defined as physically inactive.

### Frailty

All participants underwent frailty assessments using the FRAIL Scale, conducted by trained research coordinators. This tool, developed by the Geriatric Advisory Panel of the International Academy of Nutrition and Aging, serves as a widely applicable screening measure suitable for administration by various healthcare professionals and caregivers (19). The FRAIL Scale encompasses five distinct domains, namely: Fatigue, Resistance, Ambulation, Illnesses, and Loss of Weight. In the context of this scale, “Fatigue” is defined as a subjective experience of persistent tiredness for “most or all” of the past 4 weeks. “Resistance” receives a positive score if the patient encounters difficulty climbing 10 steps without the aid of an assistive device or if they need to stop and rest during the process. Similarly, “Ambulation” receives a positive score if the patient faces challenges when attempting to walk independently for 2 blocks. “Illnesses” is deemed positive if the patient presents with five or more co-morbidities, including hypertension, diabetes, cancer, asthma, chronic lung disease, heart attack, congestive heart failure, angina, arthritis, stroke, and kidney disease. Lastly, “Loss of Weight” registers as positive if the patient has experienced a body weight reduction exceeding 5% over the past year. Each positive domain is assigned a score of 1. The score range for this scale is 0–5 points, The higher the score, the greater the degree of frailty.

### Physical and social support

The variable of “physical and social support” was measured using the Social Support Scale developed by Taiwanese scholar Huangjia and Chen (20) as a Chinese version. It was later optimized by mainland Chinese scholar Duan (21) to suit the measurement of social support for PA among the older population in mainland China (Supplementary material 2). This scale has since been validated in several dissertations by mainland scholars and has shown good reliability and validity (22). This scale is specifically designed to assess the support received by older adults for their physical activity behaviors. It includes four dimensions: family support, friend support, informational support, and instrumental support, comprising a total of 17 assessment items. It effectively evaluates the physical and social support required for the physical activity behaviors of the older adult. In this scale, which served as an independent variable, a 5-point Likert scale was employed. Responses ranged from “completely disagree” to “completely agree”, with scores ranging from 1 to 5. The total score ranges from 17 to 105, with higher scores indicate a higher level of physical and social support. In the present study, the Cronbach alpha coefficients for this scale ranged from 0.83 to 0.91 for each support factor assessed among the participants.

### Physical activity motivation

To investigate PA motivation in older adults with SCD, all participants completed the Shorten Motives for Physical Activity Measure-Revised (Shorten MPAM-R) questionnaire. The Motives for Physical Activity Measure-Revised (MPAM-R) was originally developed by Richard et al. in 1997 (23), comprising 30 items. To better suit the Chinese context, Chinese scholar Chen Shanping introduced this scale and conducted a simplified revision, resulting in the Shorten MPAM-R (24). The Shorten MPAM-R employs a 5-point Likert scale, ranging from 1 (lowest motivation) to 5 (highest motivation), to assess the extent to which personal motives. It has 15 items and includes 5 subscales: fitness or the desire to be healthy (3 items), appearance or physical attractiveness (3 items), competence or skill acquisition (3 items), social interactions or meeting people (3 items), and enjoyment or having fun (3 items). The total score ranges from 5 to 75, with higher scores indicating greater motivation and lower scores indicating lower motivation. This scale has been validated in the Chinese population, including adolescents and older adults, demonstrating good reliability and validity. In this study, the Cronbach's alpha coefficients for this scale ranged from 0.79 to 0.85 for each motivational factor assessed among the participants.

### Fall history

In this study, the fall history specifically refers to the occurrences of falling within the past year. Patients were queried about falls within the previous year, with an affirmative response denoting a positive history of falls, and a negative response indicating an absence of such incidents.

## Ethnical considerations

Participants were informed that their participation was voluntary, and they had the option to withdraw at any time. Each participant provided written informed consent. The study followed strict confidentiality guidelines. Approval for this research was obtained from the institutional review board. Ethics clearance (2023SJL57) was granted by the ethics committee of the corresponding author's university.

## Data analysis

The data were analyzed using SPSS 27.0 and AMOS 23.0 (IBM Corp., Armonk, NY, USA). Firstly, descriptive statistics, such as frequencies and percentages, were employed to characterize participants' sociodemographic attributes. Secondly, descriptive statistics, like mean ± SD, were used to outline frailty, PA motivation, physical and social support, and PA behavior. Thirdly, Pearson correlation tests were conducted to examine associations among the independent variables, dependent variables, mediators, and moderators. Fourthly, a path model was estimated to investigate both the direct effects of the relationship between frailty scores, physical and social support, and PA behavior, as well as their indirect effects through PA motivation. The path model was also added the control variables (gender, marital status, place of living, educational attainment, current smoker, current drinking, and personal monthly income). In the path analysis, direct effects of the independent variables (frailty scores, physical, and social support) on the dependent variable (PA behavior) were estimated. Additionally, indirect effects of the independent variables (frailty scores, physical, and social support) on the dependent variable (PA behavior) through the mediating variables (PA motivation) were also estimated. The total and specific indirect effects were computed using bootstrapping with 5,000 samples. Model fit indices for path analysis included the χ^2^-test, root mean square error of approximation (RMSEA) ≤ 0.08, comparative fit index (CFI) ≥0.95, and Tucker Lewis index (TLI) ≥0.95 (25). Finally, the moderation effects of whether fall history were analyzed separately between frailty score and PA motivation and between frailty and PA behavior using the process v4.1 macro (26). To mitigate non-essential multicollinearity and enhance the interpretability of the results, centralization of frailty was performed, and interaction terms between the independent variable and moderator variables were computed.

## Results

### Characteristics of the participants

The average age of the 289 participants was 67.0 years (SD = 4.8), with 32.5% of them aged 70 years or older. More than half of the participants were male (57.4%), 21.2% were either unmarried or widowed, and the majority (49.8%) had an educational level of primary school or below. 69.9% of the participants resided in rural areas, 34.9% were current smokers, and 27.3% were current alcohol consumers. Nearly half of the participants had a monthly personal income in the range of 2,000–3,000 yuan (Table 1).

**Table 1:** Characteristics of participants (*n* = 289).

**Variables**	**Frequency (%)**
**Gender**	
Male	166 (57.4)
Female	123 (42.6)
**Marital status**	
Married/living with partner	228 (78.9)
Unmarried/widowed	61 (21.1)
**Place of living**	
Rural area	202 (69.9)
Urban area	87 (30.1)
**Educational attainment**	
Primary school and below	144 (49.8)
Middle school	98 (33.9)
High school and above	47 (16.3)
**Current smoker**	
No	188 (65.1)
Yes	101 (34.9)
**Current drinking**	
No	210 (72.7)
Yes	79 (27.3)
**Personal monthly income (RMB)**	
≤ 1,000	48 (16.6)
1,000 <RMB ≤ 2,000	49 (17.0)
2,000 <RMB ≤ 3,000	133 (46.0)
>3,000	59 (20.4)

### Prevalence of physical activity behavior and correlation with variables

Among the 289 participants, 117 (40.4%) exhibited physical inactivity behavior (didn't meet the WHO guidelines). Their average frailty score was 2.26 (SD = 1.50), the average score for physical and social support was 52.55 (SD = 13.24), and the average score for PA motivation was 49.81 (SD = 10.32). Additionally, 41 participants (14.1%) reported experiencing falls in the past year. Table 2 presents statistically significant associations among the key variables. Specifically, there was a negative correlation coefficient of −0.193 between physical activity and frailty, which was significant at *P* < 0.01. There was a positive correlation coefficient of 0.274 between physical activity and social support, also significant at *P* < 0.01. The correlation coefficient between physical activity and exercise motivation was 0.410, indicating a positive relationship that was significant at *P* < 0.01. Finally, there was a negative correlation coefficient of −0.392 between physical activity behavior and falls, which was also significant at *P* < 0.01.

**Table 2:** Correlations among main variables (*n* = 289).

	**Frailty**	**Physical and social support**	**PA motivation**	**PA behavior**	**Fall history**
Frailty	1				
Physical and social support	−0.07	1			
PA motivation	−0.233^**^	0.201^**^	1		
PA behavior	−0.193^**^	0.274^**^	0.410^**^	1	
Fall history	−0.202^**^	−0.250^**^	−0.272^**^	−0.392^**^	1
Mean	2.260	52.550	49.806	0.600	-
SD	1.499	13.237	10.319	0.492	-

PA, Physical activity; SD, standard deviation.

^**^P <0.01.

### Testing the proposed model and path analysis

The fit indices of the structural equation model are as follows: χ^2^/df = 1.145, which is <5; RMSEA = 0.022, which is <0.08; CFI = 0.988 and TLI = 0.984, both >0.95. These results indicate that the fit indices of the structural equation model meet the required criteria, making it suitable for structural equation modeling analysis.

The path coefficients in the structural equation model indicate the relationships between variables, while the critical ratio (C.R.) is used to assess the significance of these coefficients. Generally, a C.R. value equal to or >1.96 is considered indicative of significance at the 0.05 level. Table 3 displays the standardized regression coefficients and variance parameter estimates for our study. The path coefficient between frailty and PA motivation is −0.220, with a C.R. value of −3.902, indicating a highly significant negative impact (*p* < 0.001). For physical and social support and PA motivation, the path coefficient is 0.186, with a C.R. value of 3.297, signifying a highly significant positive impact (*p* < 0.001). Similarly, the path coefficient between PA motivation and PA behavior is 0.370, with a C.R. value of 7.676, indicating a highly significant positive relationship (*p* < 0.001). Additionally, the path coefficient between physical and social support and PA behavior is 0.131, with a C.R. value of 2.795, suggesting a significant positive effect (*p* < 0.01). Finally, the path coefficient between frailty and PA behavior is −0.115, with a C.R. value of −2.436, indicating a significant negative impact (*p* < 0.05).

**Table 3:** Path coefficients between variables (*n* = 289).

**Path**			**β**	***b***	**S.E**.	**C.R**.	***P*-value**
PA motivation	<—	Frailty	−0.22	−1.515	0.388	−3.902	<0.001
PA motivation	<—	Physical and social support	0.186	0.145	0.044	3.297	<0.001
PA behavior	<—	PA motivation	0.370	0.018	0.002	7.676	<0.001
PA behavior	<—	Physical and social support	0.131	0.005	0.002	2.795	0.005
PA behavior	<—	Frailty	−0.115	−0.038	0.015	−2.436	0.015

PA, Physical activity; S.E., Standard error; C.R., Critical ratio.

### Mediation analysis

We conducted a mediation analysis using the bootstrap method with 5,000 resamples to calculate 95% confidence intervals. The results for the two mediation pathways are shown in Table 4.

**Table 4:** Mediation analysis (*n* = 289).

**Path**	**Effect decomposition**	**Point estimate**	**S.E**.	**Lower 95%CI**	**Higher 95%CI**	***P*-value**
Frailty → PA behavior	Mediation effect	−0.027	0.008	−0.043	−0.015	0.001
	Direct effect	−0.038	0.016	−0.065	−0.013	0.015
	Total effect	−0.064	0.018	−0.095	−0.036	0.001
Physical and social support → PA behavior	Mediation effect	0.003	0.001	0.001	0.004	0.001
	Direct effect	0.005	0.002	0.002	0.008	0.014
	Total effect	0.007	0.002	0.004	0.011	0.002

PA, Physical activity; S.E., Standard error; CI, Confidence interval.

Firstly, regarding the pathway from frailty to exercise motivation and then to physical activity, both the lower and upper intervals show that the effect is not zero, and the *p*-values are <0.01. This means that the mediation effect is significant. Frailty has a noticeable impact on physical activity behavior (β = −0.064; 95% CI = −0.095 to −0.036; *p* < 0.001). About 58.5% of this impact is direct (β = −0.038; 95% CI = −0.065 to −0.013; *p* = 0.015), and the remaining 41.5% is mediated through exercise motivation (β = −0.027; 95% CI = −0.043 to −0.015; *p* = 0.001).

Secondly, for the pathway from social support to exercise motivation and then to physical activity, both the lower and upper intervals indicate that the effect is not zero, and the *p*-values are <0.01. This confirms the significance of the mediation effect. Physical and social support has a noticeable impact on physical activity behavior (β = 0.008; 95% CI = 0.004–0.011; *p* = 0.002). About 62.5% of this impact is direct (β = 0.005; 95% CI = 0.002–0.008; *p* = 0.014), while the remaining 37.5% is mediated through exercise motivation (β = 0.003; 95% CI = 0.001–0.004; *p* = 0.001).

### Moderation analysis

To analyze the moderating effect of fall history between frailty and PA motivation, to reduce non-essential multicollinearity and enhance interpretability, we centered frailty and calculated interaction terms between the independent variable and the moderating variable. In Model 1 (Table 5), fall history (*B* = −8.844, *t* = −4.610, *p* < 0.001) had a significant negative impact on PA motivation, and frailty (*B* = −1.924, *t* = −5.006, *p* < 0.001) also had a significant negative impact on PA motivation. However, the interaction term between fall history and frailty (*B* = 1.133, *t* = 0.801, *p* > 0.05) had no significant effect on PA motivation, indicating that fall history did not act as a moderator between frailty and PA motivation.

**Table 5:** Moderating effect of fall history (*n* = 289).

**Variables**	**Model 1**^**a**^**: Frailty**→**PA motivation**→**PA behavior**		
	***B***	**S.E**.	***t***
Frailty	−1.924	0.384	−5.006^***^
Fall history	−8.844	1.919	−4.610^***^
Frailty^*^Fall history	1.133	1.415	0.801
*R*	0.466		
*R* ^2^	0.217		
*F*	6.985		
	**Model 2**^a^**: Frailty**→**PA behavior**		
Frailty	−1.176	0.236	−4.987^***^
Fall history	−10.284	2.750	−3.739^***^
Frailty^*^Fall history	−4.269	1.465	−2.914^**^
−2LL	247.856		
CoxSnell	0.389		
Nagelkrk	0.525		

PA, physical activity; S.E., Standard error.

^a^Adjusted for age, gender, marital status, place of living, educational attainment, current smoker, current drinking, and personal monthly income.

^**^P <0.01.

^***^P <0.001.

In Model 2 (Table 5), fall history (*B* = −10.284, *Z* = −3.739, *p* < 0.001) had a significant negative impact on PA behavior, and frailty (*B* = −1.176, *Z* = −4.987, *p* < 0.001) also had a significant negative impact on PA behavior. Moreover, the interaction term between fall history and frailty (*B* = −4.269, *Z* = −2.914, *p* < 0.001) had a significant impact on PA behavior, indicating that fall history acted as a moderator between frailty and PA behavior.

**Figure 2:**
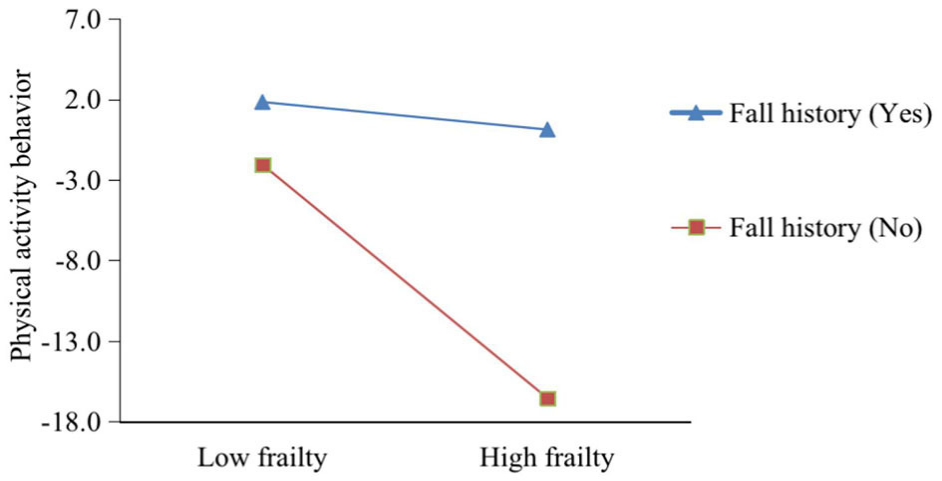
The moderating role of fall history in the relationship between frailty and physical activity behavior.

To further understand the moderating effect of fall history between frailty and PA behavior, we conducted a simple slope test using Aiken and West's (27) method, grouping frailty based on one standard deviation above and below the mean. Further simple slope analysis revealed (see Figure 2) that when fall history was present (simple slope = −5.700, *Z* = −4.906, *p* < 0.001), frailty had a significant negative predictive effect on PA behavior, and when fall history was absent (simple slope = −4.840, *Z* = −3.210, *p* < 0.01), frailty still had a significant negative predictive effect on PA behavior.

## Discussion

This study proposed a research model based on COM-B to examine the factors link to PA behavior in older adults with SCD through specific pathways. Firstly, frailty had both a direct influence on PA behavior and an indirect impact through PA motivation. Secondly, physical and social support had direct effects on PA behavior, also mediated by PA motivation. These relationships revealed two significant direct effects and two specific indirect effects. Additionally, the influence of frailty on PA behavior was moderated by a history of falls.

The relatively high prevalence of physically inactive behavior among older adults with SCD, as observed in this study at 40.4%, is a concerning finding that merits discussion. Currently, there have been no study direct measurements of PA levels in the older SCD population. However, many previous studies have investigated PA levels in older populations, and our research has found that the prevalence of physical inactivity in the older SCD group is significantly higher than in the normal older population. Chan et al. (28) conducted a study involving 3,969 Malaysians aged 60 and above, assessing their physical activity behavior. They found that nearly one-third (29.8%) of Malaysian older adults were physically inactive. Similarly, Pengpid and Peltzer (29) conducted a survey of physical activity behavior among 72,262 middle-aged and older adult residents in Indian communities, revealing that 36.7% of individuals were physically inactive. However, when compared to the group afflicted with cognitive impairment, the older SCD population in this study exhibited lower levels of physical inactivity. For example, Miller et al. (30) conducted research on 1,875 community-dwelling participants aged 65 and above with cognitive impairment, finding that 56% of participants with Cognitive Impairment No Dementia (CIND) were physically inactive. Therefore, the older SCD population, as a critical window, represents an important period for physical activity intervention. As cognitive abilities decline further, the prevalence of physical inactivity in this group may increase.

Previous studies have investigated the relationship between PA and frailty, consistently finding a negative correlation (31, 32), which aligns with the results of this study. However, in our research, we considered frailty as an independent variable, representing a specific capability of older adults with SCD and found that older SCD individuals with higher frailty scores were more likely to exhibit physical inactivity. To be precise, the relationship between frailty and PA may involve a reciprocal interaction. Researchers such as Sagong et al. (33) observed significant reciprocal relationships between frailty and high physical activity among middle-aged Koreans (70–79 years old). Similarly, Ding et al. (34) found that as individuals age, the impact of frailty on changes in activity limitations becomes more pronounced. Furthermore, frailty exerts significant indirect effects through reduced PA, depressive symptoms, and cognitive impairment. A study Omura et al. (35) conducted on middle-aged and older adults with SCD in the United States also pointed out that 49.2% of adults with SCD reported functional limitations, and this proportion increased as their PA levels decreased. Therefore, when the frailty scores of older adults with SCD are higher, it indicates that certain functions are limited, and their capacity for PA is diminished. Consequently, the results of this study emphasize the importance of screening for frailty in older adults with SCD. On one hand, frailty screening is cost-effective, for instance, by utilizing the FRAIL scale, which is a quick and convenient tool (36). On the other hand, frailty may serve as a significant barrier to their PA. Thus, comprehensive interventions, especially tailored PA programs, can be implemented within the frail population.

In this study, we observed significant differences in the impact of physical and social support on promoting PA behavior in older adults with SCD. Older adults with SCD who reported higher levels of physical and social support were less likely to be physically inactive. Prior research has underscored the importance of social support in promoting PA among older populations. Studies by Zimmer and McDonough (37) delved into nine forms of social support to identify the strongest predictor of PA in older adults. Their findings revealed that larger social networks, increased social contact with network members, and participation in community-related activities were associated with greater engagement in physical activities. Patterson et al. (38) conducted interviews with 14 women aged 65 and older and found that social support facilitated positive body image management among older women, increasing their motivation for PA. Additionally, research by Schlenk et al. (39) suggested that individuals who perceived support for their self-reported PA tended to experience greater improvements in daily minutes of PA compared to those who did not perceive such support. It is important to note that quantitative studies specifically examining the quantitative effects of physical support among older adults have been relatively limited, with many relying on qualitative interviews for insights (40). A recent umbrella review (8) also highlighted that environmental factors and resources are among the highest-rated determinants associated PA behavior in individuals with dementia/MCI. Hence, in our study, we chose to treat physical and social support as a primary variable, aligning with the comprehensive representation of the “opportunity” component in the COM-B model. This represents a distinctive feature compared to previous research and also assesses the utility of this scale. Future studies examining the support received by older adults with SCD in the context of PA may consider employing this scale for assessment.

In our study, we examined the mediating role of PA motivation in the relationships between frailty, physical and social support, and PA behavior. Our findings highlight the significant mediating effects of PA motivation in explaining these associations. Motivation is a proximal determinant of behavior, exerting a direct influence on PA behavior, a concept supported by prior research emphasizing the centrality of motivation in health behavior change interventions (41, 42). In our study, we measured PA motivation in older SCD individuals across five dimensions: desire to be healthy, appearance or physical attractiveness, competence or skill acquisition, social interactions or meeting people, and enjoyment or having fun, comprehensively covering motivational factors for engaging in physical activities among older adults. An increase in frailty levels may directly impact the competence or skill acquisition motivation of older adults with SCD, thereby indirectly affecting their PA behavior. Physical and social support are closely linked to various motivational aspects, with social influence being a prominent motivating factor according to previous research. Our research model, rooted in the COM-B framework and designed to investigate the factors associated PA behavior in older adults with SCD, contributes to the scientific understanding of this subject.

Our study also highlights a significant moderating effect of fall history on the relationship between frailty and PA behavior, which is a notable finding. Falls, as a potential indicator of physical vulnerability, have the capacity to amplify the negative impact of frailty on PA behavior. This observation aligns with previous research (43) conducted in older adults, indicating that a history of falls can exacerbate the challenges associated with physical inactivity. For instance, Park et al. (11) found that fallers exhibited lower gait performance and engaged in fewer physical activities compared to non-fallers. Compared to others, older adults with SCD who have a history of falls may encounter heightened physical limitations and a heightened fear of recurrent falls, further impeding their participation in physical activities. This moderating effect emphasizes the necessity for tailored interventions that not only address frailty but also encompass fall prevention strategies for this vulnerable population. For those with a history of falls in the older adults with SCD, intervention programs could be designed to include multifaceted exercise plans (44). Such plans could serve the dual purpose of increasing PA levels in older adults with SCD while simultaneously reducing the risk of future falls.

This study has several limitations. First, a cross-sectional design was employed, which precludes establishing causal relationships between variables. Future research should consider experimental or longitudinal designs to better examine the causal assumptions of this model. Second, the use of self-reported measures may introduce subjectivity and potential reporting biases. Third, our study focused solely on investigating the mediating role of PA motivation, while other factors may also be at play. Future research should explore a more comprehensive set of mediating factors. Finally, this study was conducted exclusively in a single county in southeastern China, which, while ensuring sample homogeneity, raises concerns regarding selection bias and potential lack of representativeness. Future research should aim to conduct investigations in multiple regions across China to enable comparative analysis and account for regional differences.

## Conclusions

Understanding factors associated with PA behavior is a key step in ensuring the promoting PA in older adults with SCD. Our study employed a comprehensive research model rooted in the COM-B framework to investigate the determinants of PA behavior among older adults with SCD. Our findings illuminated several critical pathways that shed light on the factors associated PA behavior in this population. PA motivation not only partially mediates between frailty and PA behavior but also partially mediates between physical and social support and PA behavior. These findings emphasize the multifaceted nature of PA behavior in older adults with SCD and the importance of considering frailty, motivational factors and support systems in promoting PA. Furthermore, a noteworthy aspect of our study was the identification of the moderating effect of fall history on the relationship between frailty and PA behavior. This finding underscores the significance of tailored interventions that not only target frailty but also incorporate fall prevention strategies for individuals with a history of falls.

## Data availability statement

The raw data supporting the conclusions of this article will be made available by the authors, without undue reservation.

## Ethics statement

The studies involving humans were approved by Ethics Committee of Shanxi Medical University. The studies were conducted in accordance with the local legislation and institutional requirements. The participants provided their written informed consent to participate in this study.

## Author contributions

YC: Conceptualization, Data curation, Formal analysis, Investigation, Methodology, Resources, Writing – original draft, Writing – review & editing. WL: Conceptualization, Data curation, Writing – original draft, Writing – review & editing. HY: Formal analysis, Investigation, Methodology, Resources, Supervision, Validation, Writing – review & editing.
