# Supplementary material for: Determinants of physical activity behavior among older adults with subjective cognitive decline based on the capability, opportunity, motivation, and behavior model: mediating and moderating effects
Source: Front Public Health. 2024 Jan 8;11:1338665. doi: 10.3389/fpubh.2023.1338665 (PMC10805022; doi:10.3389/fpubh.2023.1338665)
Supplement: Supplementary file 2 [file Table_2.PDF]

Supplementary file 2

社会支持量表（social support scale）

序号 (No.)	维度 (scale dimensions)	题目(items)	完全不同意 completely disagree	基本不同意 disagree	一般 neutral	基本同意 agree	完全同意 completely agree
1	家人支持 (family support)	家人会和我讨论与运动有关的事物<br>In my family, we discuss things related to exercise.					
2		家人会鼓励我运动<br>My family encourages me to exercise.					
3		家人热爱运动，促使我也热爱运动<br>My family has a passion for exercise, which motivates me to love exercise as well.					
4		家人会陪我一起运动<br>My family exercises with me.					
5	朋友支持 (friend support)	朋友热爱运动，促使我也热爱运动<br>My friends have a passion for exercise, which motivates me to love exercise as well.					
6		朋友会称赞我的运动表现<br>My friends praise my exercise performance.					
7		朋友会陪我一起运动<br>My friends exercise with me.					
8		朋友会鼓励我运动<br>My friends encourages me to exercise.					
9		朋友会和我讨论与运动有关的事物<br>My friends discuss things related to exercise with me.					
10	讯息性支持(information support)	我有机会接受到公共体育指导员的指导<br>I have the opportunity to receive guidance from public sports					

		instructors.					
11		我能通过互联网（如通过手机、电脑 上网等）获得运动锻炼的相关知识<br>I can access knowledge related to exercise and fitness through the internet, such as using a phone or computer to go online.					
12		我居住的社区经常开展运动锻炼相关的知识宣传<br>My community frequently conducts awareness campaigns related to exercise and fitness.					
13	工具性支持 (instrumental support)	我能够享受到丰富的公共体育服务设施资源<br>I have access to a wide range of public sports service facilities and resources					
14		有足够的公共体育场地可以用来运动锻炼<br>There are enough public sports facilities available for me to exercise.					
15		我有机会参加健身知识讲座<br>I have the opportunity to attend fitness knowledge lectures.					
16		我有机会参加健身竞赛活动<br>I have the opportunity to participate in fitness competitions.					
17		有与自己喜爱的运动相关的团体组织可以参加<br>There are group organizations related to my favorite exercise that I can join.					
