# Supplementary material for: Determinants of physical activity behavior among older adults with subjective cognitive decline based on the capability, opportunity, motivation, and behavior model: mediating and moderating effects
Source: Front Public Health. 2024 Jan 8;11:1338665. doi: 10.3389/fpubh.2023.1338665 (PMC10805022; doi:10.3389/fpubh.2023.1338665)
Supplement: Supplementary file 3 [file Table_3.PDF]

Supplementary file 3

Reference 20. Hong Huangjia, Chen Guixiang. A study on the relationship between social support and participation motivation of deaf athletes. Cheng Kung Journal of Physical Education. 2010;42(1):1-18.

Original source: [20]洪煌佳, 陳癸享. "聽障選手的社會支持及參與動機之關係研究." 成大體育學刊 42.1 (2010): 1-18.

Reference 21. Duan Mengshaung.The Impact of the Interaction between Social Support and Public Sports Services on the Persistence of Physical Exercise Behavior in the Elderly [Master's thesis, Hebei University]. Hebei, China: Hebei University, 2019.

Original source: 段梦双. 社会支持与公共体育服务的交互作用对老年人运动锻炼坚持行为的影响研究[D]. 河北大学, 2019.

{URL}:https://kns.cnki.net/kcms2/article/abstract?v=aGn3Ey0ZxcBH4kgWksQxjLdhm9R6dX1V6Jyn23Mq-vUZM6beeAjUVpUmF8fVYFugJWgXGRqbUIpjKvEhe7gqAB3kUMi5jJCMvfhvqwp44Sprd-uzhSHs_bEqtMN9A5peJIqaa8rng7L5PJwl41Svow==&uniplatform=NZKPT&language=CHS

Reference 22. Wang Xiaofeng. "The Influence of Social Support on Physical Exercise Behavior in the Elderly: The Mediating Role of Exercise Motivation" [Master's thesis]. Guangxi Normal University; 2023.

Original source: 王小锋. 社会支持对老年人体育锻炼行为的影响：锻炼动机的中介作用[D]. 广西师范大学, 2023.

{URL}: https://link.cnki.net/doi/10.27036/d.cnki.ggxsu.2023.000336

{DOI}: 10.27036/d.cnki.ggxsu.2023.000336
